# Prognostic value of neutrophil-to-lymphocyte ratio in human epidermal growth factor receptor 2-negative breast cancer patients who received neoadjuvant chemotherapy

**DOI:** 10.1038/s41598-020-69965-1

**Published:** 2020-08-04

**Authors:** Soong June Bae, Yoon Jin Cha, Changik Yoon, Dooreh Kim, Janghee Lee, Soeun Park, Chihwan Cha, Jee Ye Kim, Sung Gwe Ahn, Hyung Seok Park, Seho Park, Seung Il Kim, Joon Jeong

**Affiliations:** 1 Department of Surgery, Gangnam Severance Hospital, Yonsei University College of Medicine, 211 Eonju-ro, Gangnam-gu, Seoul, 06273 Republic of Korea; 2 Department of Pathology, Gangnam Severance Hospital, Yonsei University College of Medicine, Seoul, Republic of Korea; 3 Department of Surgery, St. Mary’s Hospital, The Catholic University of Korea, College of Medicine, Seoul, Republic of Korea; 4 Department of Surgery, Severance Hospital, Yonsei University College of Medicine, Seoul, Republic of Korea

**Keywords:** Cancer, Breast cancer, Cancer therapy, Tumour immunology

## Abstract

We aimed to investigate the correlation between neutrophil-to-lymphocyte ratio (NLR) and pathologic complete response (pCR) and survival outcomes in human epidermal growth factor receptor 2 (HER2)-negative breast cancer patients who received neoadjuvant chemotherapy. The baseline NLR was evaluated in non-metastatic, HER2-negative breast cancer patients who received neoadjuvant chemotherapy. Baseline NLR was calculated as absolute neutrophil per lymphocyte count from pre-treatment blood samples. Any value ≥ 2.74 was considered to be a high NLR. In the 1,097 patients studied, 272 (24.4%) had high NLR and 825 (75.6%) had low NLR. The high NLR was an independent factor for pCR (OR 0.595; 95% CIs 0.398–0.890; *P* = 0.011). Furthermore, high NLR was a significant independent parameter affecting DFS (HR 2.298; 95% CIs 1.691–3.124; *P* < 0.001) and OS (HR 1.905; 95% CIs 1.167–3.108; *P* = 0.010). Regardless of the baseline NLR, survival outcomes were excellent in patients who achieved pCR, but high NLR was associated with worse survival for patients with residual invasive disease. Our study showed that NLR was predictive for treatment response and a prognostic factor in patients with HER2-negative breast cancer who received neoadjuvant chemotherapy. Moreover, we identified that high NLR was associated with poor survival outcomes in patients who did not achieve pCR.

## Introduction

Neoadjuvant chemotherapy before definitive cancer surgery is increasingly being accepted as a treatment for breast cancer^1^. Neoadjuvant chemotherapy has survival outcomes equivalent to those of adjuvant chemotherapy^2,3^, but also has the following advantages: it can increase the rate of breast-conserving surgery by reducing the size and extent of locally advanced tumors, control occult micro-metastasis, and estimate sensitivity to treatment regimen^4,5^. Besides, it encourages the development and approval of new agents by allowing rapid assessment of drug efficacy in neoadjuvant trials^6^. To date, pathologic complete response (pCR) following the reception of neoadjuvant chemotherapy has been suggested as a surrogate marker for a long-term clinical benefit^7–9^. In particular, patients with HER2-positive breast cancer or triple-negative breast cancer (TNBC) who achieved pCR show improved survival than those with residual invasive disease^10^.

Over the past decades, it has been repeatedly reported that the host’s immune system is an essential factor in determining clinical outcomes in breast cancer^11,12^. The neutrophil-to-lymphocyte ratio (NLR), which is a peripheral blood-based parameter that can be measured easily, has been identified as a factor that reflects the host’s immune system. A high neutrophil level has been implicated to play a pivotal role in carcinogenesis and disease progression by enhancing angiogenesis, inhibiting cancer cell apoptosis, and reducing the adhesion of the extracellular matrix^13–15^. Moreover, neutrophils suppress the activated T cells, the cytolytic activity of lymphocytes, and natural killer cells^16^, whereas lymphocytes are known to up-regulate anti-cancer effects^17^. There is growing evidence that a high NLR is associated with poor response to chemotherapy^18,19^ and unfavorable prognosis in breast cancer^20–24^. However, the predictive or prognostic value of NLR in breast cancer patients who were treated with neoadjuvant chemotherapy remains controversial.

The current study aimed to investigate the relationship between pre-treatment NLR and clinical outcomes in HER2-negative breast cancer patients who received neoadjuvant chemotherapy. We explored whether NLR had a bearing on the survival outcomes of patients who attained pCR and those who did not.

## Results

### Baseline characteristics

In total, 1,097 HER2-negative breast cancer patients were studied. The median age was 47 (range 20–84). Of these patients, 825 (75.2%) were assigned to the low NLR group (NLR < 2.74) and 272 (24.8%) to the high NLR group (NLR ≥ 2.74). The baseline characteristics per the NLR status are listed in Table 1. The high NLR group contained a significantly younger population (45 vs. 48, *p* < 0.001) and higher clinical T stages and Ki-67 levels than the low NLR group (Table 1). Besides, the high NLR group received less anthracycline-cyclophosphamide followed by taxane chemotherapy than the high NLR group (Table 1).

**Table 1:** Baseline characteristics.

	NLR < 2.74 (N = 825)	NLR ≥ 2.74 (N = 272)	Total (N = 1,097)	p-value
**Age (median, range)**	48 (20–84)	45 (22–76)	47 (20–84)	< 0.001
**Histologic type**				0.407
IDC	771 (93.5%)	258 (94.9%)	1,029 (93.8%)	
Others	54 (6.5%)	14 (5.1%)	68 (6.2%)	
**ER**				0.067
Positive	483 (58.5%)	142 (52.2%)	625 (57.0%)	
Negative	342 (41.5%)	130 (47.8%)	472 (43.0%)	
**PR**				0.752
Positive	370 (44.8%)	119 (43.8%)	489 (44.6%)	
Negative	455 (55.2%)	149 (56.3%)	608 (55.4%)	
**Subgroup**				0.113
HR+HER2−	491 (59.5%)	147 (54.0%)	638 (58.2%)	
TNBC	334 (40.5%)	125 (46.0%)	459 (41.8%)	
Ki-67*				0.010
< 14	143 (34.7%)	34 (23.1%)	177 (31.7%)	
≥ 14	269 (65.3%)	113 (76.9%)	382 (68.3%)	
**cT**				0.025
1	125 (15.2%)	28 (10.3%)	153 (13.9%)	
2	558 (67.6%)	181 (66.5%)	739 (67.4%)	
3	142 (17.2%)	63 (23.2%)	205 (18.7%)	
**cN**				0.444
Negative	128 (15.5%)	37 (13.6%)	165 (15.0%)	
Positive	697 (84.5%)	235 (86.4%)	932 (85.0%)	
**pCR**				0.033
No	635 (77.0%)	226 (83.1%)	861 (78.5%)	
Yes	190 (23.0%)	46 (16.9%)	236 (21.5%)	
**Regimen**				0.009
AC-T	661 (80.1%)	196 (72.1%)	857 (78.1%)	
AC	26 (3.2%)	10 (3.7%)	36 (3.3%)	
AT	113 (13.7%)	47 (17.3%)	160 (14.6%)	
Others^**†**^	25 (3.0%)	19 (7.0%)	44 (4.0%)	

*NLR* neutrophil to lymphocyte ratio, *ER* estrogen receptor, *PR* progesterone receptor, *cT* clinical T stage, *cN* clinical N stage, *pCR* pathologic complete response, *AC-T* doxorubicin and cyclophosphamide followed by taxane, *AC* doxorubicin and cyclophosphamide, *AT* doxorubicin and taxane.

*Missing values.

^†^Others: cyclophosphamide, doxorubicin, 5-fluorouracil (CAF); cyclophosphamide, methotrexate, 5-fluorouracil (CMF); taxane; taxane plus carboplatin.

Of all patients, 638 (58.2%) were hormone receptor (HR)-positive, having HER2-negative breast cancer (HR+HER2−), and 459 (41.8%) had triple-negative breast cancer (TNBC). The patients with TNBC were younger and had higher clinical T stages and Ki-67 levels than those with HR+HER2− breast cancer (Supplementary Table 1). There was no difference in NLR between the two groups.

### pCR rate according to NLR

**Figure 1:**
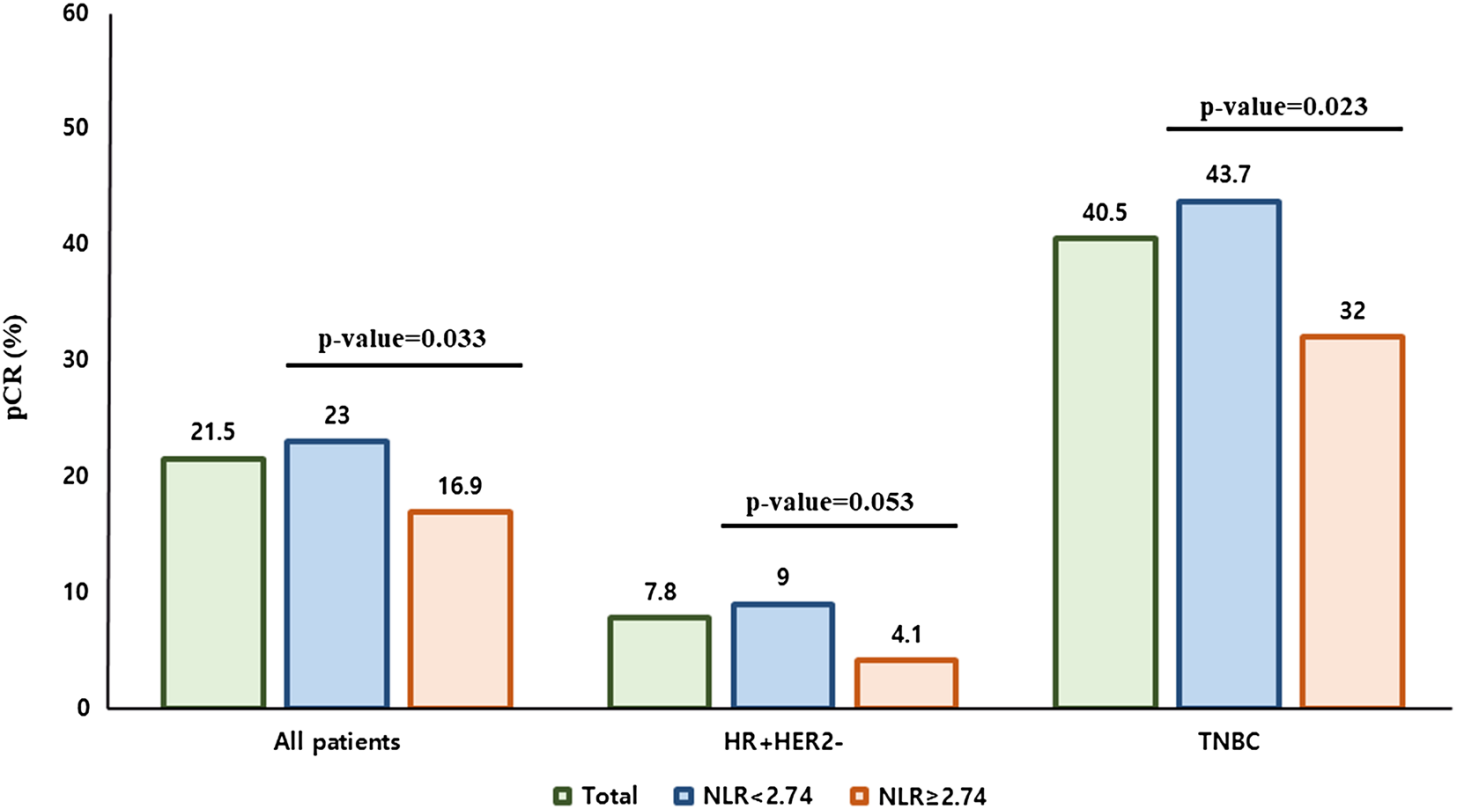
pCR rate according to baseline NLR. *pCR* pathologic complete response, *NLR* neutrophil-to-lymphocyte ratio.

Baseline NLR was associated with response to neoadjuvant systemic therapy: pCR was achieved in 190 (23.0%) of the 825 low NLR patients and in 46 (16.9%) of the 272 high NLR patients (*P* = 0.033; Fig. 1). Elevated NLR was significantly associated with low pCR in univariable analysis (OR 0.680; 95% CIs 0.476–0.971; *P* = 0.034; Table 2), and it remained a significant factor in a multivariable analysis adjusted for other clinicopathologic parameters (OR 0.595; 95% CIs 0.398–0.890; *P* = 0.011; Table 2).

**Table 2:** Odds ratios (ORs) and 95% confidential intervals (CIs) for pCR in all patients.

	Univariate		Multivariate	
	OR (95% CIs)	P-value	OR (95% CIs)	P-value
**Age**	0.996 (0.982–1.011)	0.62	0.992 (0.977–1.008)	0.336
**NLR**				
< 2.74	Ref		Ref	
≥ 2.74	0.680 (0.476–0.971)	0.034	0.595 (0.398–0.890)	0.011
**cT stage**		0.002		< 0.001
1	Ref		Ref	
2	1.320 (0.856–2.036)	0.209	0.804 (0.489–1.324)	0.392
3	0.596 (0.336–1.056)	0.076	0.327 (0.172–0.621)	0.001
**cN stage**				
Negative	Ref		Ref	
Positive	0.407 (0.285–0.582)	< 0.001	0.583 (0.385–0.883)	0.011
**ER**				
Positive	Ref		Ref	
Negative	7.713 (5.462–10.892)	< 0.001	5.017 (3.033–8.299)	< 0.001
**PR**				
Positive	Ref		Ref	
Negative	7.515 (5.034–11.220)	< 0.001	2.283 (1.289–4.044)	0.005
**Regimen**		0.002		< 0.001
AC-T	Ref		Ref	
AC	0.516 (0.198–1.345)	0.176	0.407 (0.142–1.168)	0.095
AT	0.381 (0.225–0.644)	< 0.001	0.308 (0.174–0.544)	< 0.001
Others*	0.941 (0.457–1.939)	0.870	0.741 (0.333–1.651)	0.463

*pCR* pathologic complete response, *NLR* neutrophil to lymphocyte ratio, *cT* clinical T stage, *cN* clinical N stage, *ER* estrogen receptor, *PR* progesterone receptor, *AC-T* doxorubicin and cyclophosphamide followed by taxane, *AC* doxorubicin and cyclophosphamide, *AT* doxorubicin and taxane.

*Others: cyclophosphamide, doxorubicin, 5-fluorouracil (CAF); cyclophosphamide, methotrexate, 5-fluorouracil (CMF); taxane; taxane plus carboplatin.

In the HR+HER2− breast cancer patients, pCR was achieved in 44 (9.0%) of 491 low NLR patients and in 6 (4.1%) of 147 high NLR patients (*P* = 0.053; Fig. 1). In the multivariable analysis, the patients with elevated NLR tended have low pCR rates, but this was not statistically significant (OR 0.433; 95% CIs 0.178–1.056; *P* = 0.066; Supplementary Table 2). In TNBC patients, pCR was achieved in 146 (43.7%) of 334 low NLR patients and in 40 (32.0%) of 125 high NLR patients (*P* = 0.023; Fig. 1). The multivariable analysis showed that high NLR tended to be associated with low pCR rate, although it was not statistically significant (OR 0.645; 95% CIs 0.408–1.020; *P* = 0.061; Supplementary Table 2).

### Survival outcomes according to NLR

**Figure 2:**
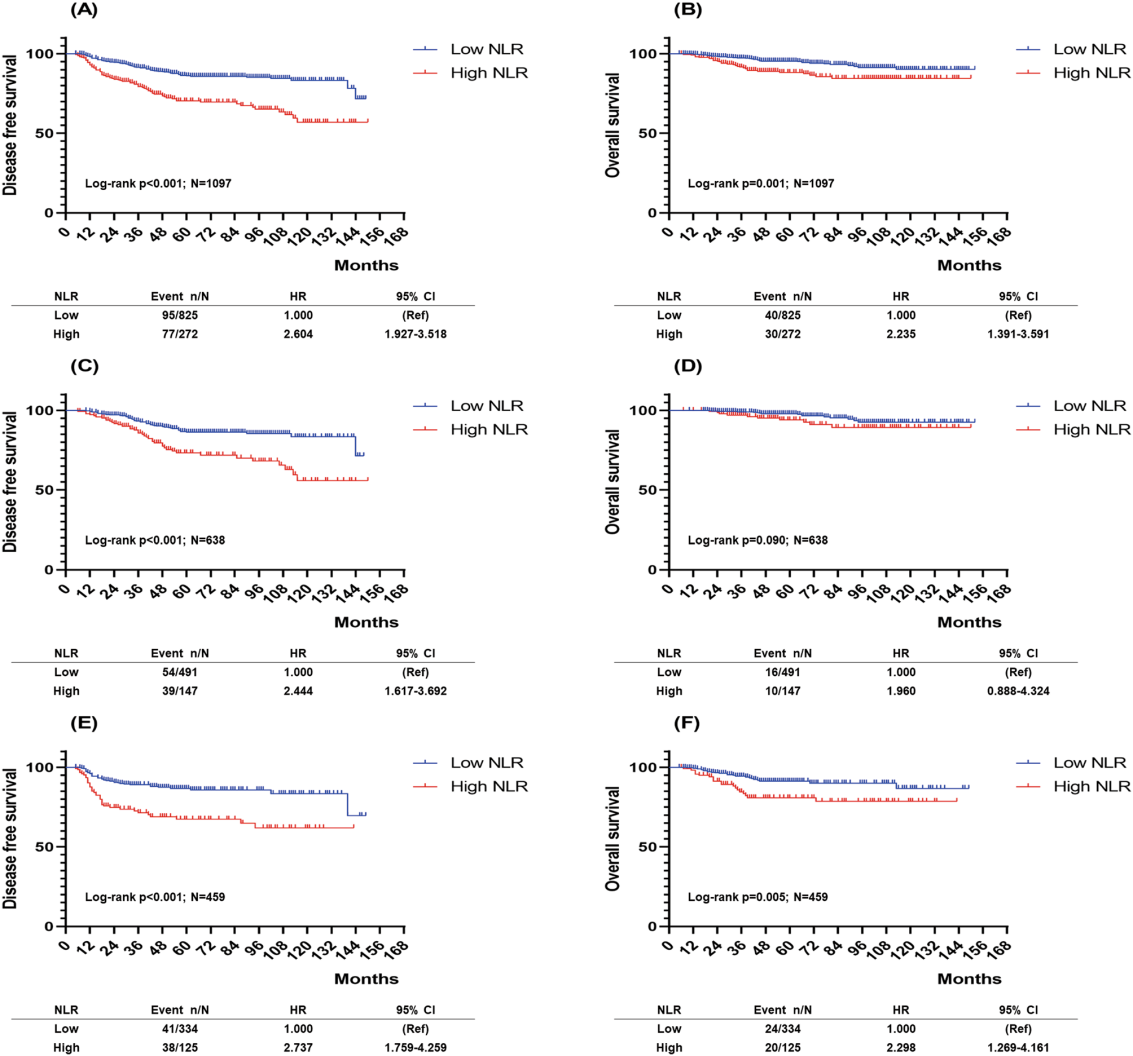
Prognostic ability of the baseline NLR. Kaplan–Meier curves of (**A**) DFS in all patients, (**B**) OS in all patients, (**C**) DFS in HR+HER2− breast cancer, (**D**) OS in HR+HER2− breast cancer, (**E**) DFS in TNBC, (**F**) OS in TNBC, *DFS* disease-free survival, *OS* overall survival, *HR* hormone receptor, *HER2* human epidermal growth factor receptor 2, *TNBC *triple-negative breast cancer. All graphs were prepared using the software Graphpad Prism Version 8 (GraphPad Software, USA, https://www.graphpad.com/scientific-software/prism/).

During the median follow up period of 56 months (range 5–152 months), patients with low NLR had a significantly longer DFS and OS than those with high NLR (Fig. 2A,B). In HR+HER2− breast cancer patients, those with low NLR had a significantly longer DFS than those with high NLR, although there was no difference in OS between both groups (Fig. 2C,D). In contrast, in TNBC patients, those with low NLR had significantly longer DFS and OS than those with high NLR (Fig. 2E,F).

In both univariable and multivariable analyses, the baseline NLR was an independent prognostic factor for DFS (HR 2.604; 95% CIs 1.927–3.518; *P* < 0.001, HR 2.298; 95% CIs 1.691–3.124; *P* < 0.001, respectively; Table 3) and OS (HR 2.235; 95% CIs 1.391–3.591; *P* = 0.001, HR 1.905; 95% CIs 1.167–3.108; *P* = 0.010, respectively; Table 3). Similarly, in TNBC, the univariable and multivariable analyses showed that the baseline NLR was an independent prognostic factor for DFS (HR 2.737; 95% CIs 1.759–4.259; *P* < 0.001, HR 2.491; 95% CIs 1.599–3.882; *P* < 0.001, respectively; Supplementary Table 3) and OS (HR 2.298; 95% CIs 1.269–4.161; *P* = 0.006, HR 2.053; 95% CIs 1.132–3.725; *P* = 0.018, respectively; Supplementary Table 3). In HR+HER2− breast cancer, the baseline NLR was significantly associated with DFS in univariable analysis (HR 2.444; 95% CIs 1.617–3.692; *P* < 0.001) and multivariable analysis (HR 2.323; 95% CIs 1.537–3.511; *P* < 0.001; Supplementary Table 3), but not with OS (Supplementary Table 3).

**Table 3:** Hazard ratios (HRs) and 95% confidential intervals (CIs) for disease-free survival and overall survival in all patients.

Variables	Disease-free survival				Overall survival			
	Univariate		Multivariate		Univariate		Multivariate	
	HRs (95% CIs)	P-value	HRs (95% CIs)	P-value	HRs (95% CIs)	P-value	HRs (95% CIs)	P-value
**Age**	0.988 (0.972–1,003)	0.120	0.989 (0.974–1.005)	0.179	1.017 (0.993–1.042)	0.160	1.013 (0.989–1.038)	0.294
**NLR**								
< 2.74	Ref		Ref		Ref		Ref	
≥ 2.74	2.604 (1.927–3.518)	< 0.001	2.298 (1.691–3.124)	< 0.001	2.235 (1.391–3.591)	0.001	1.905 (1.167–3.108)	0.010
**cT stage**		0.042		0.247		0.108		0.216
1	Ref		Ref		Ref		Ref	
2	1.093 (0.701–1.702)	0.696	0.996 (0.634–1.565)	0.986	0.933 (0.479–1.818)	0.839	0.767 (0.389–1.513)	0.444
3	1.687 (1.014–2.807)	0.044	1.354 (0.805–2.276)	0.253	1.683 (0.793–3.573)	0.175	1.254 (0.582–2.703)	0.564
**cN stage**								
Negative	Ref		Ref		Ref		Ref	
Positive	1.706 (1.003–2.900)	0.049	1.752 (1.014–3.027)	0.044	5.037 (1.232–20.587)	0.024	5.302 (1.279–21.986)	0.022
**ER**								
Positive	Ref		Ref		Ref		Ref	
Negative	1.642 (1.217–2.215)	0.001	2.197 (1.375–3.511)	0.001	3.856 (2.310–6.435)	< 0.001	5.457 (2.463–12.092)	< 0.001
**PR**								
Positive	Ref		Ref		Ref		Ref	
Negative	1.323 (0.976–1.794)	0.071	1.034 (0.649–1.648)	0.889	2.334 (1.389–3.923)	0.001	0.930 (0.422–2.048)	0.857
**pCR**								
Yes	Ref		Ref		Ref		Ref	
No	3.658 (2.034–6.579)	< 0.001	4.553 (2.483–8.348)	< 0.001	2.765 (1.197–6.387)	0.017	4.020 (1.695–9.535)	0.002
**Regimen**		0.033		0.723		0.110		0.914
AC-T	Ref		Ref		Ref		Ref	
AC	1.307 (0.574–2.975)	0.523	1.252 (0.546–2.868)	0.596	1.132 (0.274–4.679)	0.864	0.920 (0.219–3.866)	0.910
AT	1.625 (1.134–2.328)	0.008	1.189 (0.822–1.721)	0.357	1.922 (1.133–3.260)	0.015	1.189 (0.681–2.075)	0.543
Others	1.736 (0.933–3.231)	0.082	1.262 (0.672–2.373)	0.469	1.524 (0.545–4.259)	0.422	0.882 (0.308–2.529)	0.815

*NLR* neutrophil to lymphocyte ratio, *cT* clinical T stage, *cN* clinical N stage, *ER* estrogen receptor, *PR* progesterone receptor, *pCR* pathologic complete response, *AC-T* doxorubicin and cyclophosphamide followed by taxane, *AC* doxorubicin and cyclophosphamide, *AT* doxorubicin and taxane.

*Others: cyclophosphamide, doxorubicin, 5-fluorouracil (CAF); cyclophosphamide, methotrexate, 5-fluorouracil (CMF); taxane; taxane plus carboplatin.

### Survival outcomes according to NLR and pCR

**Figure 3:**
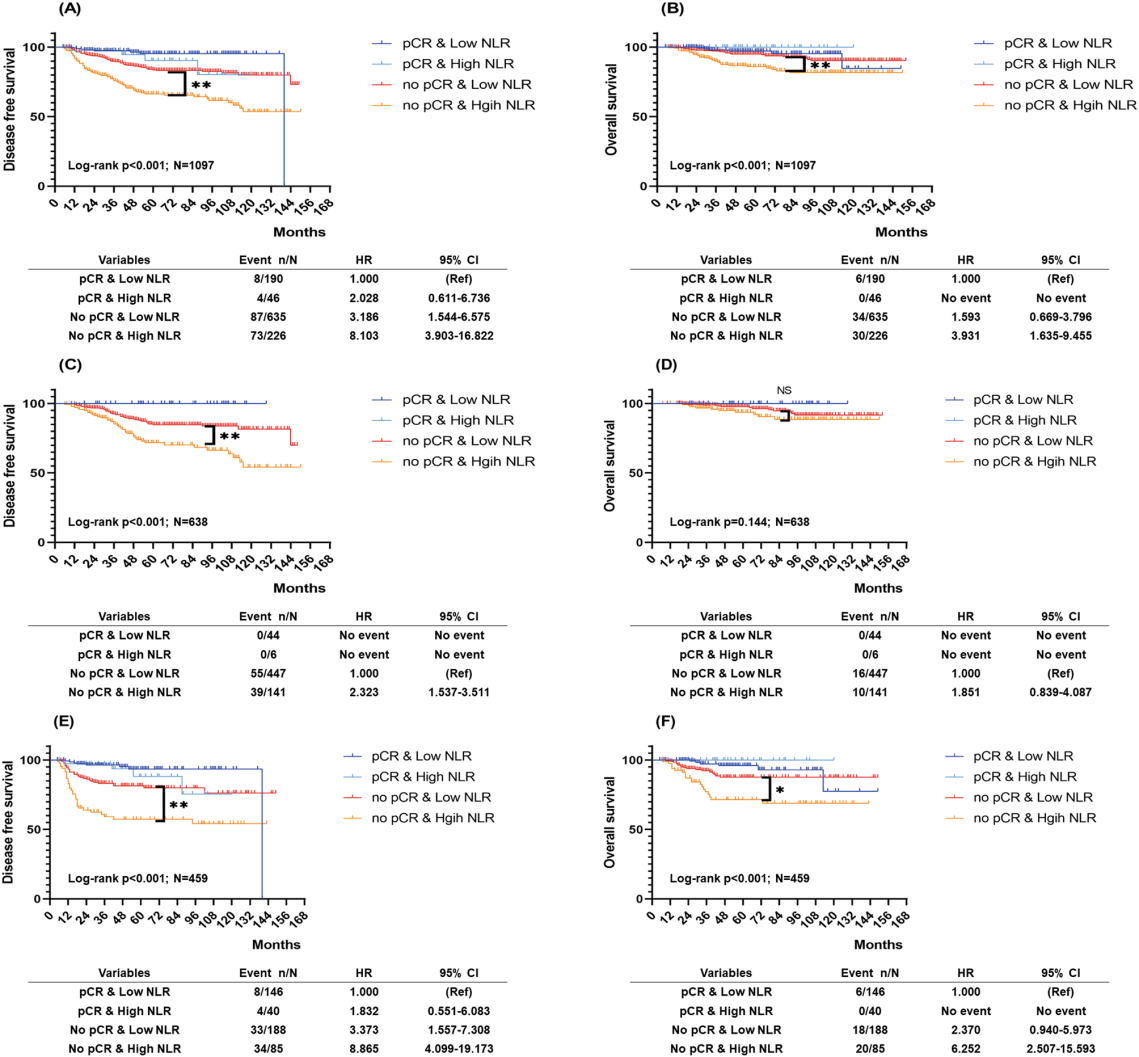
Prognostic ability of the combined with pCR and baseline NLR. Kaplan–Meier curves of (**A**) DFS in all patients, (**B**) OS in all patients, (**C**) DFS in HR+HER2− breast cancer, (**D**) OS in HR+HER2− breast cancer, (**E**) DFS in TNBC, (**F**) OS in TNBC. *DFS* disease-free survival, *OS* overall survival, *HR* hormone receptor, *HER2* human epidermal growth factor receptor 2, *TNBC* triple-negative breast cancer, *NS* not significant. *P < 0.05; **P < 0.001. All graphs were prepared using the software Graphpad Prism Version 8 (GraphPad Software, USA, https://www.graphpad.com/scientific-software/prism/).

Our results also showed that patients with pCR had better survival outcomes than those with residual invasive disease (Supplementary Fig. 1). When combined NLR and pCR analysis was performed, patients who achieved pCR had better survival rates in both HR+HER2− breast cancer and TNBC cases than those with residual invasive disease, regardless of baseline NLR status. Notably, the survival rates varied significantly in patients with residual invasive disease based on baseline NLR status. We were able to identify the patients with high NLR and residual invasive disease as a high-risk group who had a worse DFS (Fig. 3A), and OS (Fig. 3B) than the other groups. In HR+HER2− patients, those with high NLR and residual invasive disease were significantly associated with poor DFS (Fig. 3C), but not OS (Fig. 3D). In TNBC patients, those with high NLR and residual invasive disease had adverse DFS (Fig. 3E), and OS (Fig. 3F).

## Discussion

Recently, high tumor-infiltrating lymphocytes (TILs) has been identified as biomarker related to pCR and better clinical outcomes in patients with breast cancer who received neoadjuvant chemotherapy^25^. These data suggest that the immune system might have an important role in terms of treatment response and prognosis. Similar to TILs, we found that the NLR was an independent factor for pCR and survival in patients with HER2-negative breast cancer who received neoadjuvant chemotherapy. Nevertheless, the precise mechanism underlying the impact of NLR on chemotherapy response and clinical outcomes in breast cancer has been unclear. Previous studies speculated several potential mechanisms. The high NLR can be caused by neutrophilia and/or lymphopenia linked to the inflammatory response and depletion of anti-tumor immune function, which in turn leads to tumor progression^26–28^. Few data revealed that the high NLR was associated with increased peri-tumoral macrophages, which contribute to chemotherapy resistance^29,30^. In addition, anthracycline and taxane-based chemotherapy that most patients received in this study provide an anti-cancer immune response via triggering immunogenic cell death^31^, which may affect the difference in chemotherapy efficacy according to NLR. Further studies to verify this issue are warranted.

The differences in pCR and survival according to baseline NLR were more pronounced in TNBC subype than in HR+HER2− breast cancer subtype. The previous studies demonstrated that TNBC was more immunogenic compared to other subtypes of breast cancer, and higher pre-treatment TILs were correlated with increased pCR rates, and improved survival in TNBC patients treated with neoadjuvant chemotherapy^25,32^. Also, the adjuvant chemotherapy trials in TNBC have shown that TILs are strongly associated with improved survival^33,34^. Similar to our findings, several lines of evidence described that the pre-treatment NLR was predictive of the efficacy of neoadjuvant chemotherapy and disease outcomes in TNBC^19,35,36^. Compared to these recent relatively small-cohort studies of about a hundred patients with TNBC, our research has the advantage of having analyzed a larger cohort.

Unlike in TNBC, the predictive and prognostic role of immune-related markers, including TIL and NLR, remain unclear in HR+HER2− breast cancer^20–22^. Furthermore, a pooled analysis described that the patients with high TIL were inversely associated with survival in HR+HER2− breast cancer^25^. However, most of the previous studies that investigated the prognostic role of NLR in HR+HER2− breast cancer had the limitation of using small cohorts and short follow up periods of less than 5 years. In contrast, the current study assessed a relatively large number of HR+HER2− breast cancer patients who received neoadjuvant chemotherapy and discovered that low NLR was related to favorable survival rates in terms of DFS. Given that disease-related events occur steadily after 5 years of diagnosis in HR+HER2− breast cancer patients^37^, and the median follow up period was about 5 years in this study, further research with longer term follow up is needed to validate these findings.

It is well known that the prognosis is poor in patients with residual tumor after neoadjuvant therapy^10^. The CREATE-X and KATHERINE trials have already demonstrated that new therapeutic approaches, such as the addition of further chemotherapy or the change of HER2-targeted therapy, conferred a survival benefit in patients with residual invasive disease after neoadjuvant therapy^38,39^. Besides, in our study, baseline NLR provided an additional prognostic information in patients with residual disease after neoadjuvant chemotherapy in line with previous study^40^: Even when limited to patients with residual invasive disease after neoadjuvant chemotherapy, high NLR was related to poor survival outcomes. These results suggest that patients with poor treatment response and an impaired immune system should be considered as a high-risk group for adverse prognosis, and there is a need for a new therapeutic strategy to improve survival outcomes in these patients.

The major limitation of our study is its retrospective nature. Particularly, the histologic grade, lymphovascular invasion, and Ki-67 levels, which could be factors associated with pCR and prognosis, were not routinely evaluated in pre-treatment biopsy samples. Although the Ki-67 was estimated in about half of whole cohort, and NLR was still an independent factor related to pCR, DFS, and OS in these patients (Supplementary Table 4 and 5). Also, we could not analyze the relationship between NLR and TILs, which represent the immune system in the tumor microenvironment. Currently, NLR cutoff value has not been established, and the acceptable discriminant point varies across studies. In this analysis, NLR cutoff point was set at 2.74, which is within the 1.8–4.0 range reported by the previous studies^19–22^, and this value should be validated by independent cohorts.

In conclusion, baseline NLR may be a potential biomarker of host immunity for predicting response to neoadjuvant chemotherapy and prognosis in HER2-negative breast cancer patients. Furthermore, we identified that elevated baseline NLR was associated with worse clinical outcomes among patients with residual invasive disease after neoadjuvant chemotherapy. Our findings suggest that new therapeutic strategies are needed to improve survival in these patients.

## Methods

### Study population

We retrospectively analyzed the data of non-metastatic, HER2-negative breast cancer patients who received neoadjuvant chemotherapy followed by surgery between January 2007 to June 2018 at Gangnam Severance Hospital and Severance Hospital. Medical records were reviewed to collect patient data like age and clinicopathologic data such as medical history, estrogen receptor (ER) status, progesterone receptor (PR) status, human epidermal growth factor 2 (HER2) status, Ki-67 levels, clinical T stage, clinical N stage, pCR, and laboratory data. The clinical stages were determined per the 7th edition of the American Joint Committee on Cancer guidelines.

**Figure 4:**
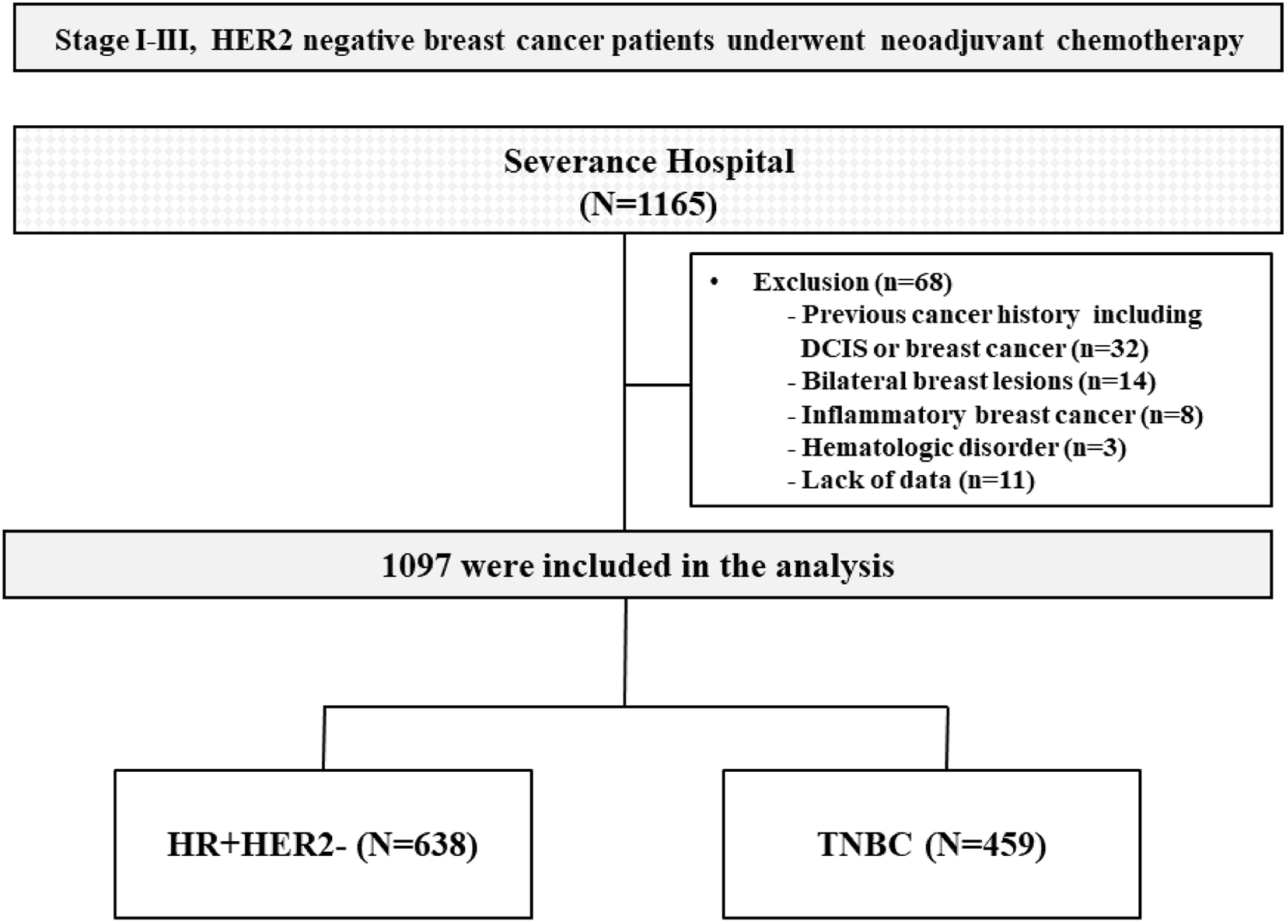
Study population.

The exclusion criteria for this study were: (i) a history of cancer including ductal carcinoma in situ or invasive breast cancer, and other malignancies, (ii) presence of bilateral breast lesions, (iii) presence of inflammatory breast cancer, (iv) lack of patient information such as baseline NLR, and (v) presence of a hematologic disorder or systemic inflammatory disease. Eventually, 1,097 patients were studied (Fig. 4) and of this number, 1,017 (92.3%) received anthracycline-taxane-based chemotherapy. The details chemotherapy regimens are given in Table 1.

Following the Good Clinical Practice guidelines and the Declaration of Helsinki, our study was approved by the Institutional Review Board at Severance Hospital, Yonsei University, Seoul, Republic of Korea (Local IRB number: 3-2018-0191). The need for informed consent was waived under the approval of the IRB due to the retrospective design.

### Baseline neutrophil-to-lymphocyte ratio (NLR)

Baseline NLR was calculated as neutrophil count per lymphocyte count from blood samples taken within 2 weeks before initiation of neoadjuvant chemotherapy. The cutoff point of 2.74 was decided as the maximum area under curve value using receiver operating characteristics curve for predicting disease-free survival (DFS); hence, patients were assigned into two groups: the high NLR group (≥ 2.74) and the low NLR group (< 2.74). An about a quarter of our cohort were on this basis classified as high NLR.

### Pathology

In our immunohistochemistry (IHC) study, core needle biopsy samples were stained using appropriate antibodies specific for four markers: ER (1:100 dilution, clone 6F11; Novocastra, Newcastle upon Tyne, UK), PR (clone 16; Novocastra, UK), HER2 (4B5 rabbit monoclonal antibody; Ventana Medical Systems, Tucson, AZ, USA), and Ki-67 (MIB-1; Dako, Glostrup, Denmark). ER- and PR-positive were defined as a cutoff value of ≥ 1% positively stained nuclei^41^, or according to the modified Allred system: positive, Allred scores 2–8,and negative, Allred scores 0^42^. The HER2 status was defined as positive with a score of 3+, and negative with a score of 0 or 1+. Tumors with scores of 2+ were sent for fluorescent in situ hybridization analysis according to the protocol given by the supplier (PathVysion kit; Vysis, Downers Grove, IL, USA, or HER2 inform; Ventana)^43^. Staining positive for the nuclear antigen Ki-67 was evaluated in a quantitatively and visually way using light microscopes, and the positive tumor cell percentage was reported as the Ki-67 labeling index (LI)^44^. We considered Ki-67 levels ≥ 14% as high^45^.

### Statistical analysis

Continuous variables were compared using Student’s *t*-test, and categorical variables were compared by the Chi-square test or Fisher’s exact test. The pCR was defined as no evidence of invasive cancer residue in both breast and axillary lymph nodes (ypT0/is, ypN0) based on the pathologic evaluation of the surgical specimen after neoadjuvant chemotherapy^46^. Multivariable analysis for pCR was performed using a binary logistic regression model. Odds ratios (ORs) and 95% confidence intervals (CIs) with two-sided *P*-values were given. DFS was measured as the period between breast cancer diagnosis and first tumor recurrence, including locoregional recurrence, and distant recurrence. The overall survival (OS) was measured as the period between breast cancer diagnosis to death by any cause. The Kaplan–Meier method was used to calculate DFS and OS, and the results between groups were compared using the log-rank test. Multivariable analysis for survival outcomes was carried out using a Cox proportional hazards model. All analyses were performed using SPSS version 23 (SPSS; Chicago, IL, USA), and statistical significance was defined as *P*-value < 0.05.

### Consent for publication

All authors have given consent for publication.

## Supplementary information

Supplementary Information.

## Data Availability

The datasets generated and analyzed during the current study are available from the corresponding author on request.

## References

[1] Senkus E (2015). Primary breast cancer: ESMO clinical practice guidelines for diagnosis, treatment and follow-up. Ann. Oncol..

[2] Mauri D, Pavlidis N, Ioannidis JP (2005). Neoadjuvant versus adjuvant systemic treatment in breast cancer: a meta-analysis. J. Natl. Cancer Inst..

[3] van der Hage JA (2001). Preoperative chemotherapy in primary operable breast cancer: results from the European Organization for Research and Treatment of Cancer trial 10902. J. Clin. Oncol..

[4] Sachelarie I (2006). Primary systemic therapy of breast cancer. Oncologist.

[5] Mayer EL, Carey LA, Burstein HJ (2007). Clinical trial update: implications and management of residual disease after neoadjuvant therapy for breast cancer. Breast Cancer Res..

[6] Prowell TM, Pazdur R (2012). Pathological complete response and accelerated drug approval in early breast cancer. N. Engl. J. Med..

[7] Bonadonna G, Valagussa P, Brambilla C, Ferrari L (1993). Preoperative chemotherapy in operable breast cancer. Lancet.

[8] Liedtke C (2008). Response to neoadjuvant therapy and long-term survival in patients with triple-negative breast cancer. J. Clin. Oncol..

[9] Mieog JS, van der Hage JA, van de Velde CJ (2007). Preoperative chemotherapy for women with operable breast cancer. Cochrane Database Syst. Rev..

[10] Cortazar P (2014). Pathological complete response and long-term clinical benefit in breast cancer: the CTNeoBC pooled analysis. Lancet.

[11] Savas P (2016). Clinical relevance of host immunity in breast cancer: from TILs to the clinic. Nat. Rev. Clin. Oncol..

[12] Andre F (2013). Molecular pathways: involvement of immune pathways in the therapeutic response and outcome in breast cancer. Clin. Cancer Res..

[13] De Larco JE, Wuertz BR, Furcht LT (2004). The potential role of neutrophils in promoting the metastatic phenotype of tumors releasing interleukin-8. Clin. Cancer Res..

[14] Queen MM, Ryan RE, Holzer RG, Keller-Peck CR, Jorcyk CL (2005). Breast cancer cells stimulate neutrophils to produce oncostatin M: potential implications for tumor progression. Cancer Res..

[15] Wculek SK, Malanchi I (2015). Neutrophils support lung colonization of metastasis-initiating breast cancer cells. Nature.

[16] Yamanaka T (2007). The baseline ratio of neutrophils to lymphocytes is associated with patient prognosis in advanced gastric cancer. Oncology.

[17] Niederhuber JE (1997). Cancer vaccines: the molecular basis for T cell killing of tumor cells. Oncologist.

[18] Chen Y (2016). Pretreatment neutrophil-to-lymphocyte ratio is correlated with response to neoadjuvant chemotherapy as an independent prognostic indicator in breast cancer patients: a retrospective study. BMC Cancer.

[19] Asano Y (2016). Predictive value of neutrophil/lymphocyte ratio for efficacy of preoperative chemotherapy in triple-negative breast cancer. Ann. Surg. Oncol..

[20] Ethier JL, Desautels D, Templeton A, Shah PS, Amir E (2017). Prognostic role of neutrophil-to-lymphocyte ratio in breast cancer: a systematic review and meta-analysis. Breast Cancer Res..

[21] Noh H, Eomm M, Han A (2013). Usefulness of pretreatment neutrophil to lymphocyte ratio in predicting disease-specific survival in breast cancer patients. J. Breast Cancer.

[22] Liu X (2017). Prognostic role of pretreatment neutrophil to lymphocyte ratio in breast cancer patients: a meta-analysis. Medicine (Baltimore).

[23] Miyagawa Y (2020). Baseline neutrophil-to-lymphocyte ratio and c-reactive protein predict efficacy of treatment with bevacizumab plus paclitaxel for locally advanced or metastatic breast cancer. Oncotarget.

[24] Guo W (2019). Prognostic value of neutrophil-to-lymphocyte ratio and platelet-to-lymphocyte ratio for breast cancer patients: an updated meta-analysis of 17079 individuals. Cancer Med..

[25] Denkert C (2018). Tumour-infiltrating lymphocytes and prognosis in different subtypes of breast cancer: a pooled analysis of 3771 patients treated with neoadjuvant therapy. Lancet Oncol..

[26] Templeton AJ (2014). Prognostic role of neutrophil-to-lymphocyte ratio in solid tumors: a systematic review and meta-analysis. J. Natl. Cancer Inst..

[27] Quail DF, Joyce JA (2013). Microenvironmental regulation of tumor progression and metastasis. Nat. Med..

[28] Mantovani A, Allavena P, Sica A, Balkwill F (2008). Cancer-related inflammation. Nature.

[29] Harimoto N (2019). Prognostic significance of neutrophil-lymphocyte ratio in resectable pancreatic neuroendocrine tumors with special reference to tumor-associated macrophages. Pancreatology.

[30] Larionova I (2019). Interaction of tumor-associated macrophages and cancer chemotherapy. Oncoimmunology.

[31] Galluzzi L, Buque A, Kepp O, Zitvogel L, Kroemer G (2015). Immunological effects of conventional chemotherapy and targeted anticancer agents. Cancer Cell.

[32] Denkert C (2010). Tumor-associated lymphocytes as an independent predictor of response to neoadjuvant chemotherapy in breast cancer. J. Clin. Oncol..

[33] Adams S (2014). Prognostic value of tumor-infiltrating lymphocytes in triple-negative breast cancers from two phase III randomized adjuvant breast cancer trials: ECOG 2197 and ECOG 1199. J. Clin. Oncol..

[34] Loi S (2019). Tumor-infiltrating lymphocytes and prognosis: a pooled individual patient analysis of early-stage triple-negative breast cancers. J. Clin. Oncol..

[35] Patel DA (2019). Neutrophil-to-lymphocyte ratio as a predictor of survival in patients with triple-negative breast cancer. Breast Cancer Res. Treat..

[36] Pistelli M (2015). Pre-treatment neutrophil to lymphocyte ratio may be a useful tool in predicting survival in early triple negative breast cancer patients. BMC Cancer.

[37] Pan H (2017). 20-Year risks of breast-cancer recurrence after stopping endocrine therapy at 5 years. N. Engl. J. Med..

[38] von Minckwitz G (2019). Trastuzumab emtansine for residual invasive HER2-positive breast cancer. N. Engl. J. Med..

[39] Masuda N (2017). Adjuvant capecitabine for breast cancer after preoperative chemotherapy. N. Engl. J. Med..

[40] Muñoz-Montaño W (2020). Prognostic value of the pretreatment neutrophil-to-lymphocyte ratio in different phenotypes of locally advanced breast cancer during neoadjuvant systemic treatment. Clin. Breast Cancer.

[41] Hammond ME (2010). American Society of Clinical Oncology/College of American Pathologists guideline recommendations for immunohistochemical testing of estrogen and progesterone receptors in breast cancer. J. Clin. Oncol..

[42] Harvey JM, Clark GM, Osborne CK, Allred DC (1999). Estrogen receptor status by immunohistochemistry is superior to the ligand-binding assay for predicting response to adjuvant endocrine therapy in breast cancer. J. Clin. Oncol..

[43] Wolff AC (2018). Human epidermal growth factor receptor 2 testing in breast cancer: American Society of Clinical Oncology/College of American Pathologists clinical practice guideline focused update. J. Clin. Oncol..

[44] Dowsett M (2011). Assessment of Ki67 in breast cancer: recommendations from the International Ki67 in Breast Cancer working group. J. Natl. Cancer Inst..

[45] Goldhirsch A (2011). Strategies for subtypes—dealing with the diversity of breast cancer: highlights of the St. Gallen International Expert Consensus on the Primary Therapy of Early Breast Cancer 2011. Ann. Oncol..

[46] Sharma, P., Connolly, R. M., Roussos Torres, E. T. & Thompson, A. Best foot forward: neoadjuvant systemic therapy as standard of care in triple-negative and HER2-positive breast cancer. In *American Society of Clinical Oncology Educational Book. American Society of Clinical Oncology. Annual Meeting* **40**, 1–16 (2020). 10.1200/EDBK_281381 32315235

