# Supplementary material for: Prognostic value of neutrophil-to-lymphocyte ratio in human epidermal growth factor receptor 2-negative breast cancer patients who received neoadjuvant chemotherapy
Source: Sci Rep. 2020 Aug 4;10:13078. doi: 10.1038/s41598-020-69965-1 (PMC7403312; doi:10.1038/s41598-020-69965-1)

**Prognostic value of neutrophil-to-lymphocyte ratio in human epidermal growth factor receptor 2-negative breast cancer patients who received neoadjuvant chemotherapy**

Soong June Bae, MD^1^, Yoon Jin Cha, MD, PhD^2^, Changik Yoon, MD^3^, Dooreh Kim, MD^1^, Janghee Lee, MD^1^, Soeun Park, MD^1^, Chihwan Cha, MD^1^, Jee Ye Kim, MD^4^, Sung Gwe Ahn, MD, PhD^1^, Hyung Seok Park, MD, PhD^4^, Seho Park, MD, PhD^4^, Seung Il. Kim, MD, PhD^4^, and Joon Jeong, MD, PhD^1^

^1^Department of Surgery, Gangnam Severance Hospital, Yonsei University College of Medicine, Seoul, Republic of Korea;

^2^Department of Pathology, Gangnam Severance Hospital, Yonsei University College of Medicine, Seoul, Republic of Korea;

^3^Department of Surgery, St. Mary’s Hospital, The Catholic University of Korea, College of Medicine, Seoul, Republic of Korea

^4^Department of Surgery, Severance Hospital, Yonsei University College of Medicine, Seoul, Republic of Korea

**Supplementary Table 1. Baseline characteristics according to subgroup**

|  | **HR+HER2- (N=638)** | **TNBC (N=459)** | **Total (N=1097)** | **p-value** |
| --- | --- | --- | --- | --- |
| **Age (median, range)** | 48 (20-84) | 45 (22-76) | 47 (20-84) | <0.001 |
| **Histologic type** |  |  |  | 0.166 |
| IDC | 593 (92.9%) | 436 (95.0%) | 1029 (93.8%) |  |
| Others | 45 (7.1%) | 23 (33.8%) | 68 (6.2%) |  |
| **NLR** |  |  |  | 0.113 |
| NLR<2.74 | 491 (77.0%) | 334 (72.8%) | 825 (75.2%) |  |
| NLR≥2.74 | 147 (23.0%) | 125 (27.2%) | 272 (24.8%) |  |
| **Ki-67*** |  |  |  | 0.019 |
| <14 | 147 (44.3%) | 30 (13.2%) | 177 (31.7%) |  |
| ≥14 | 185 (55.7%) | 197 (86.8%) | 382 (68.3%) |  |
| **cT** |  |  |  | 0.022 |
| 1 | 114 (17.9%) | 39 (8.5%) | 153 (13.9%) |  |
| 2 | 406 (63.6%) | 333 (72.5%) | 739 (67.4%) |  |
| 3 | 118 (18.5%) | 87 (19.0%) | 205 (18.7%) |  |
| **cN** |  |  |  | <0.001 |
| negative | 66 (10.3%) | 99 (21.6%) | 165 (15.0%) |  |
| positive | 572 (89.7%) | 360 (78.4%) | 932 (85.0%) |  |
| **pCR** |  |  |  | <0.001 |
| No | 588 (92.2%) | 273 (59.5%) | 861 (78.5%) |  |
| Yes | 60 (7.8%) | 186 (40.5%) | 236 (21.5%) |  |
| **Regimen** |  |  |  | 0.100 |
| AC-T | 506 (79.3%) | 351 (76.5%) | 857 (78.1%) |  |
| AC | 23 (3.6%) | 13 (2.8%) | 36 (3.3%) |  |
| AT | 91 (14.3%) | 69 (15.0%) | 160 (14.6%) |  |
| Others† | 18 (2.8%) | 26 (5.7%) | 44 (4.0%) |  |

^*^Missing values;

NLR, neutrophil to lymphocyte ratio; cT, clinical T stage; cN, clinical N stage; pCR, pathologic complete response; AC-T, Doxorubicin and Cyclophosphamide followed by Taxane; AC, Doxorubicin and Cyclophosphamide; AT, Doxorubicin and Taxane

† Others: Cyclophosphamide, Doxorubicin, 5-Fluorouracil (CAF); Cyclophosphamide, Methotrexate, 5-Fluorouracil (CMF); Taxane; Taxane plus Carboplatin

**Supplementary Table 2. Odds ratios (ORs) and 95% confidential intervals (CIs) for pCR in HR+HER2- breast cancer and TNBC**

| **Subgroup** | **Variables** | **Univariate** | | **Multivariate** | |
| --- | --- | --- | --- | --- | --- |
|  |  | **OR (95% CIs)** | **P-value** | **OR (95% CIs)** | **P-value** |
| **HR+HER2-** | **Age** | 0.981 (0.950-1.013) | 0.238 | 0.973 (0.942-1.006) | 0.111 |
|  | **NLR** |  |  |  |  |
|  | <2.74 | Ref |  | Ref |  |
|  | ≥2.74 | 0.436 (0.182-1.046) | 0.063 | 0.433 (0.178-1.056) | 0.066 |
|  | **cT stage** |  | 0.852 |  | 0.787 |
|  | 1 | Ref |  | Ref |  |
|  | 2 | 0.890 (0.423-1.870) | 0.758 | 0.790 (0.366-1.705) | 0.548 |
|  | 3 | 0.756 (0.287-1.990) | 0.572 | 0.726 (0.269-1.957) | 0.527 |
|  | **cN stage** |  |  |  |  |
|  | negative | Ref |  | Ref |  |
|  | positive | 0.421 (0.200-0.888) | 0.023 | 0.403 (0.182-0.892) | 0.025 |
|  | **Regimen** |  | 0.184 |  | 0.173 |
|  | AC-T | Ref |  | Ref |  |
|  | AC | 0.455 (0.060-3.450) | 0.446 | 0.287 (0.036-2.314) | 0.241 |
|  | AT | 0.225 (0.054-0.943) | 0.041 | 0.246 (0.058-1.044) | 0.057 |
|  | Others* | 0.588 (0.077-4.521) | 0.610 | 0.704 (0.090-5.498) | 0.738 |
| **TNBC** | **Age** | 1.001 (0.984-1.019) | 0.899 | 0.999 (0.981-1.018) | 0.946 |
|  | **NLR** |  |  |  |  |
|  | <2.74 | Ref |  | Ref |  |
|  | ≥2.74 | 0.607 (0.392-0.940) | 0.025 | 0.645 (0.408-1.020) | 0.061 |
|  | **cT stage** |  | <0.001 |  | <0.001 |
|  | 1 | Ref |  | Ref |  |
|  | 2 | 0.760 (0.391-1.476) | 0.418 | 0.728 (0.365-1.453) | 0.368 |
|  | 3 | 0.248 (0.110-0.560) | 0.001 | 0.219 (0.095-0.507) | <0.001 |
|  | **cN stage** |  |  |  |  |
|  | negative | Ref |  | Ref |  |
|  | positive | 0.595 (0.380-0.931) | 0.023 | 0.644 (0.399-1.038) | 0.071 |
|  | **Regimen** |  |  |  |  |
|  | AC-T | Ref |  | Ref |  |
|  | AC | 0.543 (0.164-1.796) | 0.317 | 0.476 (0.135-1.677) | 0.248 |
|  | AT | 0.339 (0.184-0.624) | 0.001 | 0.319 (0.170-0.598) | <0.001 |
|  | Others* | 0.647 (0.281-1.490) | 0.306 | 0.713 (0.299-1.703) | 0.447 |

pCR, pathologic complete response; HR, hormone receptor; TNBC, triple negative breast cancer; NLR; neutrophil to lymphocyte ratio; cT, clinical T stage; cN, clinical N stage; AC-T, Doxorubicin and Cyclophosphamide followed by Taxane; AC, Doxorubicin and Cyclophosphamide; AT, Doxorubicin and Taxane

*Others: Cyclophosphamide, Doxorubicin, 5-Fluorouracil (CAF); Cyclophosphamide, Methotrexate, 5-Fluorouracil (CMF); Taxane; Taxane plus Carboplatin

**Supplementary Table 3. Hazard ratios (HRs) and 95% confidential intervals (CIs) for disease-free survival and overall survival in HR+HER2- breast cancer and TNBC**

| **Subgroup** | **Variables** | **Disease-free survival** | | | | **Overall survival** | | | |
| --- | --- | --- | --- | --- | --- | --- | --- | --- | --- |
|  |  | **Univariate** | | **Multivariate** | | **Univariate** | | **Multivariate** | |
|  |  | **HRs (95% CIs)** | **P-value** | **HRs (95% CIs)** | **P-value** | **HRs (95% CIs)** | **P-value** | **HRs (95% CIs)** | **P-value** |
| **HR+HER2-** | **Age** | 0.988 (0.965-1.011) | 0.311 | - | - | 1.014 (0.971-1.058) | 0.531 | - | - |
|  | **NLR** |  |  |  |  |  |  |  |  |
|  | <2.74 | Ref |  | Ref |  | Ref |  | Ref |  |
|  | ≥2.74 | 2.444 (1.617-3.692) | <0.001 | 2.323 (1.537-3.511) | <0.001 | 1.960 (0.888-4.324) | 0.096 | 1.893 (0.836-4.289) | 0.126 |
|  | **cT stage** |  | 0.093 |  |  |  | 0.537 |  |  |
|  | 1 | Ref |  | - | - | Ref |  | - | - |
|  | 2 | 1.071 (0.620-1.848) | 0.806 | - | - | 0.833 (0.320-2.169) | 0.708 | - | - |
|  | 3 | 1.791 (0.948-3.383) | 0.073 | - | - | 1.435 (0.461-4.463) | 0.533 | - | - |
|  | **cN stage** |  |  |  |  |  |  |  | - |
|  | Negative | Ref |  | - | - | Ref |  | - | - |
|  | Positive | 1.498 (0.654-3.429) | 0.339 | - | - | 22.929 (0.040-13190.651) | 0.334 | - | - |
|  | **pCR** |  |  |  |  |  |  |  |  |
|  | Yes | Ref |  | - | - | Ref |  | - | - |
|  | No | 23.367 (1.043-523.298) | 0.047 | - | - | 23.474 (0.075-7368.903) | 0.282 | - | - |
|  | **Regimen** |  | 0.016 |  |  |  | 0.300 |  | 0.563 |
|  | AC-T | Ref |  | - | - | Ref |  | Ref |  |
|  | AC | 0.806 (0.197-3.305) | 0.764 | - | - | - | - | - | - |
|  | AT | 1.899 (1.187-3.037) | 0.007 | - | - | 2.217 (0.981-5.008) | 0.056 | 1.839 (0.797-4.240) | 0.153 |
|  | Others* | 2.524 (1.084-5.874) | 0.032 | - | - | 1.433 (0.189-10.864) | 0.728 | 1.374 (0.181-10.439) | 0.759 |
| **TNBC** | **Age** | 0.989 (0.969-1.010) | 0.302 | - | - | 1.015 (0.987-1.044) | 0.285 | - | - |
|  | **NLR** |  |  |  |  |  |  |  |  |
|  | <2.74 | Ref |  | Ref |  | Ref |  | Ref |  |
|  | ≥2.74 | 2.737(1.759-4.259) | <0.001 | 2.491 (1.599-3.882) | <0.001 | 2.298 (1.269-4.161) | 0.006 | 2.053 (1.132-3.725) | 0.018 |
|  | **cT stage** |  | 0.257 |  | 0.653 |  | 0.135 |  |  |
|  | 1 | Ref |  | - | - | Ref |  | - | - |
|  | 2 | 0.942 (0.427-2.077) | 0.881 | - | - | 0.651 (0.250-1.692) | 0.378 | - | - |
|  | 3 | 1.363(0.565-3.285) | 0.490 | - | - | 1.279 (0.449-3.640) | 0.645 | - | - |
|  | **cN stage** |  |  |  |  |  |  |  |  |
|  | Negative | Ref |  | Ref |  | Ref |  | Ref |  |
|  | Positive | 2.154 (1.074-4.320) | 0.031 | 1.867 (0.930-3.751) | 0.079 | 5.684 (1.374-23.518) | 0.016 | 4.851 (1.170-20.105) | 0.029 |
|  | **pCR** |  |  |  |  |  |  |  |  |
|  | Yes | Ref |  | Ref |  | Ref |  | Ref |  |
|  | No | 4.172 (2.255-7.719) | <0.001 | 3.709 (1.999-6.880) | <0.001 | 4.523 (1.910-10.709) | 0.001 | 3.880 (1.633-9.220) | 0.002 |
|  | **Regimen** |  | 0.409 |  |  |  | 0.445 |  |  |
|  | AC-T | Ref |  | - | - | Ref |  | - | - |
|  | AC | 2.145 (0.776-5.929) | 0.141 | - | - | 1.924 (0.457-8.102) | 0.372 | - | - |
|  | AT | 1.343 (0.760-2.37) | 0.310 | - | - | 1.696 (0.845-3.406) | 0.137 | - | - |
|  | Others* | 1.217 (0.485-3.055) | 0.676 | - | - | 1.264 (0.382-4.181) | 0.701 | - | - |

NLR, neutrophil to lymphocyte ratio; HR, hormone receptor; TNBC, triple negative breast cancer; NLR; cT, clinical T stage; cN, clinical N stage; HG, histologic grade, pCR, pathologic complete response; AC-T, Doxorubicin and Cyclophosphamide followed by Taxane; AC, Doxorubicin and Cyclophosphamide; AT, Doxorubicin and Taxane

*Others: Cyclophosphamide, Doxorubicin, 5-Fluorouracil (CAF); Cyclophosphamide, Methotrexate, 5-Fluorouracil (CMF); Taxane; Taxane plus Carboplatin

**Supplementary Table 4. Odds ratios (ORs) and 95% confidential intervals (CIs) for pCR in patients with pre-treatment Ki-67 information**

|  | **Univariate** | | **Multivariate** | |
| --- | --- | --- | --- | --- |
|  | **OR (95% CIs)** | **P-value** | **OR (95% CIs)** | **P-value** |
| **Age** | 0.996 (0.982-1.011) | 0.62 | 0.994 (0.973-1.016) | 0.608 |
| **NLR** |  |  |  |  |
| <2.74 | Ref |  | Ref |  |
| ≥2.74 | 0.680 (0.476-0.971) | 0.034 | 0.632 (0.374-0.989) | 0.047 |
| **cT stage** |  | 0.002 |  | 0.062 |
| 1 | Ref |  | Ref |  |
| 2 | 1.320 (0.856-2.036) | 0.209 | 1.208 (0.625-2.335) | 0.574 |
| 3 | 0.596 (0.336-1.056) | 0.076 | 0.541 (0.228-1.284) | 0.164 |
| **cN stage** |  |  |  |  |
| negative | Ref |  | Ref |  |
| positive | 0.407 (0.285-0.582) | <0.001 | 0.479 (0.260-0.882) | 0.018 |
| **ER** |  |  |  |  |
| positive | Ref |  | Ref |  |
| negative | 7.713 (5.462-10.892) | <0.001 | 2.928 (1.580-5.427) | 0.001 |
| **PR** |  |  |  |  |
| positive | Ref |  | Ref |  |
| negative | 7.515 (5.034-11.220) | <0.001 | 2.127 (1.051-4.305) | 0.036 |
| **Regimen** |  | 0.002 |  | 0.008 |
| AC-T | Ref |  | Ref |  |
| AC | 0.516 (0.198-1.345) | 0.176 | 0.140 (0.016-1.168) | 0.073 |
| AT | 0.381 (0.225-0.644) | <0.001 | 0.297 (0.124-0.712) | 0.007 |
| Others* | 0.941 (0.457-1.939) | 0.870 | 1.543 (0.599-3.976) | 0.369 |
| **Ki-67** |  |  |  |  |
| <14 | Ref |  | Ref |  |
| ≥14 | 4.280 (2.448-7.483) | <0.001 | 2.541 (1.364-4.732) | 0.003 |

pCR, pathologic complete response; NLR, neutrophil to lymphocyte ratio; cT, clinical T stage; cN, clinical N stage; ER, estrogen receptor; PR, progesterone receptor; AC-T, Doxorubicin and Cyclophosphamide followed by Taxane; AC, Doxorubicin and Cyclophosphamide; AT, Doxorubicin and Taxane

*Others: Cyclophosphamide, Doxorubicin, 5-Fluorouracil (CAF); Cyclophosphamide, Methotrexate, 5-Fluorouracil (CMF); Taxane; Taxane plus Carboplatin

**Supplementary Table 5. Hazard ratios (HRs) and 95% confidential intervals (CIs) for disease-free survival and overall survival in patients with pre-treatment Ki-67 information**

| **Variables** | **Disease-free survival** | | | | **Overall survival** | | | |
| --- | --- | --- | --- | --- | --- | --- | --- | --- |
|  | **Univariate** | | **Multivariate** | | **Univariate** | | **Multivariate** | |
|  | **HRs (95% CIs)** | **P-value** | **HRs (95% CIs)** | **P-value** | **HRs (95% CIs)** | **P-value** | **HRs (95% CIs)** | **P-value** |
| **Age** | 0.988 (0.972-1003) | 0.120 | 0.994 (0.973-1.016) | 0.614 | 1.017 (0.993-1.042) | 0.160 | 1.012 (0.979-1.046) | 0.492 |
| **NLR** |  |  |  |  |  |  |  |  |
| <2.74 | Ref |  | Ref |  | Ref |  | Ref |  |
| ≥2.74 | 2.604 (1.927-3.518) | <0.001 | 2.490 (1.636-3.790) | <0.001 | 2.235 (1.391-3.591) | 0.001 | 1.954 (1.002-3.808) | 0.049 |
| **cT stage** |  | 0.042 |  | 0.881 |  | 0.108 |  | 0.137 |
| 1 | Ref |  | Ref |  | Ref |  | Ref |  |
| 2 | 1.093 (0.701-1.702) | 0.696 | 0.983 (0.539-1.764) | 0.955 | 0.933 (0.479-1.818) | 0.839 | 0.523 (0.213-1.280) | 0.156 |
| 3 | 1.687 (1.014-2.807) | 0.044 | 1.126 (0.549-2.312) | 0.746 | 1.683 (0.793-3.573) | 0.175 | 1.029 (0.377-2.810) | 0.956 |
| **cN stage** |  |  |  |  |  |  |  |  |
| negative | Ref |  | Ref |  | Ref |  | Ref |  |
| positive | 1.706 (1.003-2.900) | 0.049 | 1.559 (0.705-3.445) | 0.272 | 5.037 (1.232-20.587) | 0.024 | 5.512 (0.745-40.799) | 0.095 |
| **ER** |  |  |  |  |  |  |  |  |
| positive | Ref |  | Ref |  | Ref |  | Ref |  |
| negative | 1.642 (1.217-2.215) | 0.001 | 1.643 (0.874-3.087) | 0.123 | 3.856 (2.310-6.435) | <0.001 | 10.415 (2.805-38.663) | <0.001 |
| **PR** |  |  |  |  |  |  |  |  |
| positive | Ref |  | Ref |  | Ref |  | Ref |  |
| negative | 1.323 (0.976-1.794) | 0.071 | 1.136 (0.604-2.134) | 0.693 | 2.334 (1.389-3.923) | 0.001 | 0.690 (0.179-2.663) | 0.591 |
| **pCR** |  |  |  |  |  |  |  |  |
| Yes | Ref |  | Ref |  | Ref |  | Ref |  |
| No | 3.658 (2.034-6.579) | <0.001 | 5.328 (2.404-11.806) | <0.001 | 2.765 (1.197-6.387) | 0.017 | 5.889 (1.761-19.690) | 0.004 |
| **Regimen** |  | 0.033 |  | 0.995 |  | 0.110 |  | 0.602 |
| AC-T | Ref |  | Ref |  | Ref |  | Ref |  |
| AC | 1.307 (0.574-2.975) | 0.523 | 0.991 (0.309-3.183) | 0.988 | 1.132 (0.274-4.679) | 0.864 | 0.769 (0.104-5.710) | 0.797 |
| AT | 1.625 (1.134-2.328) | 0.008 | 0.926 (0.519-1.651) | 0.793 | 1.922 (1.133-3.260) | 0.015 | 0.716 (0.291-1.761) | 0.466 |
| Others | 1.736 (0.933-3.231) | 0.082 | 0.979 (0.385-2.485) | 0.964 | 1.524 (0.545-4.259) | 0.422 | 0.291 (0.038-2.202) | 0.232 |
| **Ki-67** |  |  |  |  |  |  |  |  |
| <14 | Ref |  | Ref |  | Ref |  | Ref |  |
| ≥14 | 2.345 (1.385-3.969) | 0.002 | 2.044 (1.169-3.574) | 0.012 | 2.308 (1.016-5.245) | 0.046 | 1.459 (0.607-3.509) | 0.399 |

NLR, neutrophil to lymphocyte ratio; cT, clinical T stage; cN, clinical N stage; ER, estrogen receptor; PR, progesterone receptor; pCR, pathologic complete response; AC-T, Doxorubicin and Cyclophosphamide followed by Taxane; AC, Doxorubicin and Cyclophosphamide; AT, Doxorubicin and Taxane

*Others: Cyclophosphamide, Doxorubicin, 5-Fluorouracil (CAF); Cyclophosphamide, Methotrexate, 5-Fluorouracil (CMF); Taxane; Taxane plus Carboplatin

**Supplementary Figure 1. Prognostic ability of pathologic complete response (pCR).**

Kaplan-Meier curves of (A) DFS in all patients, (B) OS in all patients, (C) DFS in HR+HER2- breast cancer, (D) OS in HR+HER2- breast cancer, (E) DFS in TNBC, (F) OS in TNBC.

DFS, disease-free survival; OS, overall survival; HR, hormone receptor; HER2, human epidermal growth factor receptor 2; TNBC, triple-negative breast cancer

All graphs were prepared using the software Graphpad Prism Version 8 (GraphPad Software, USA, <http://www.graphpad.com/scientific-software/prism/>).


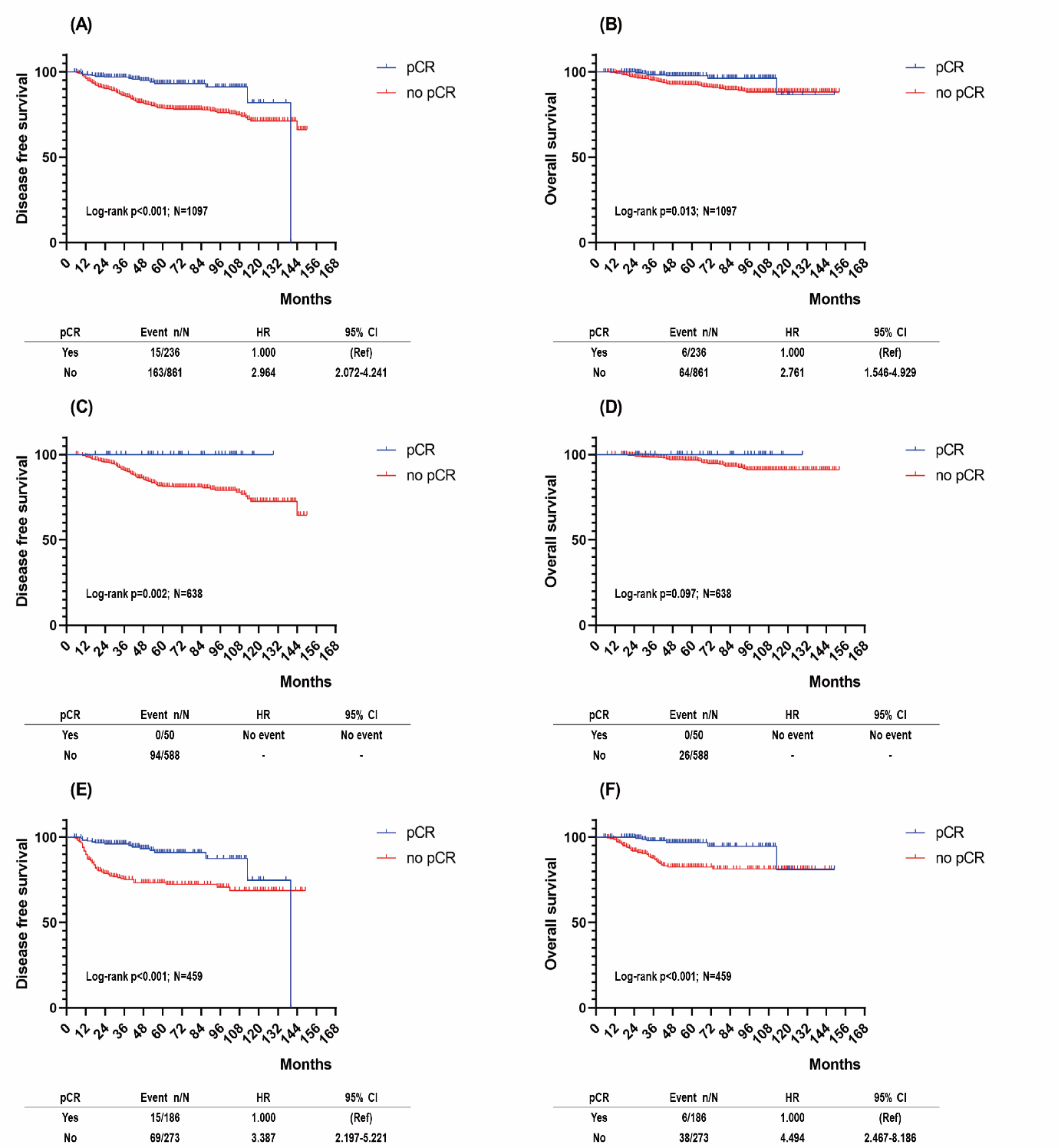

Supplement: Supplementary file 1 — Supplementary Information. [file 41598_2020_69965_MOESM1_ESM.docx]
